# Targeted Neuromodulation of Abnormal Interhemispheric Connectivity to Promote Neural Plasticity and Recovery of Arm Function after Stroke: A Randomized Crossover Clinical Trial Study Protocol

**DOI:** 10.1155/2018/9875326

**Published:** 2018-03-12

**Authors:** Michael R. Borich, Steven L. Wolf, Andrew Q. Tan, Jacqueline A. Palmer

**Affiliations:** ^1^Division of Physical Therapy, Department of Rehabilitation Medicine, Emory University, 1441 Clifton Road NE, Atlanta, GA 30322, USA; ^2^Atlanta VA Visual and Neurocognitive Center of Excellence, 1670 Clairmont Road, Decatur, GA 30333, USA

## Abstract

**Background:**

Despite intensive rehabilitation efforts, most stroke survivors have persistent functional disability of the paretic arm and hand. These motor impairments may be due in part to maladaptive changes in structural and functional connections between brain regions. The following early stage clinical trial study protocol describes a noninvasive brain stimulation approach to target transcallosally mediated interhemispheric connections between the ipsi- and contralesional motor cortices (iM1 and cM1) using corticocortical paired associative stimulation (ihPAS). This clinical trial aims to characterize ihPAS-induced modulation of interhemispheric connectivity and the effect on motor skill performance and learning in chronic stroke survivors.

**Methods/Design:**

A repeated-measures, cross-over design study will recruit 20 individuals post-stroke with chronic mild–moderate paretic arm impairment. Each participant will complete an active ihPAS and control ihPAS session. Assessments of cortical excitability and motor skill performance will be conducted prior to and at four time points following the ihPAS intervention. The primary outcome measures will be: TMS-evoked interhemispheric motor connectivity, corticomotor excitability, and response time on a modified serial reaction time task.

**Discussion/Conclusion:**

The findings from this single-site early stage clinical trial will provide foundational results to inform the design of larger-scale, multisite clinical trials to evaluate the therapeutic potential of ihPAS-based neuromodulation for upper limb recovery after stroke. This trial is registered with NCT02465034.

## 1. Background

Following a stroke, most individuals have persistent functional impairments that diminish quality of life, increase morbidity, and raise health care costs [1]. Motor impairments following stroke are the result of both direct ischemic loss of neurons combined with maladaptive brain reorganization [2]. Evidence from animal models and humans indicates that there are widespread changes in cortical network activity patterns both local and remote to the site of injury during stroke recovery [3–5]. Neuronal tissue surrounding the stroke lesion is hypoexcitable in preclinical rodent models of acute stroke [6]. This hypoexcitability is a direct result of increased levels of the inhibitory neurotransmitter, *γ*-aminobutyric acid (GABA), after stroke [7]. In rodents, pharmacological targeting of GABA levels normalized neuronal excitability and improved motor recovery [8]. In healthy individuals, reductions in cortical GABA levels accompany motor skill learning [9]. In individuals poststroke, preliminary evidence suggests movement-related GABA activity in iM1 is abnormal and correlated with arm motor impairment in stroke [10]. Specifically, abnormal activity between iM1 and cM1 via transcallosal pathways influenced by GABA-receptor activity is seen in stroke survivors [11] and associated with greater motor impairment [5]. This exaggerated interhemispheric inhibition (IHI) results in an abnormal activity imbalance between brain hemispheres. This imbalance is influenced by motor state and seems to be a primary contributor to motor impairment of the paretic arm after stroke [10, 11]. Although restoring the balance of activity between brain hemispheres has been a primary target of many novel rehabilitation strategies, limited progress has been made to improve paretic arm motor function and reduce persistent disability for stroke survivors.

One possible explanation for this limited progress in improving stroke rehabilitation outcomes is that abnormal IHI and hemispheric balance after stroke have been targeted indirectly and nonspecifically. Promising neuromodulatory rehabilitation approaches can elicit neuroplastic change using noninvasive brain stimulation (e.g., repetitive (r)TMS). However, traditional rTMS approaches target a single cortical site with limited effectiveness in restoring the balance of interhemispheric activity or meaningfully improving arm function in chronic stroke [12, 13]. The inability of rTMS to modulate the synaptic strength of pathways connecting the cM1 and iM1 may explain, in part, the equivocal efficacy of rTMS in stroke seen thus far. An alternative stimulation approach, paired associative stimulation (PAS), uses repeated pairing of TMS pulses applied over two distinct cortical sites at precise interpulse intervals. This type of corticocortical PAS is thought to induce spike-timing-dependent plasticity (STDP) that modifies the synaptic strength of specific intracortical pathways [14–16]. Critically, STDP is a key mechanism of memory formation, a prerequisite for motor skill learning that is necessary for recovery of arm function after stroke.

If STDP can be induced noninvasively by targeting direct transcallosal connections between M1s in the human brain, then this approach could offer a neuromodulation approach to restore the balance of interhemispheric activity after stroke. Using an interhemispheric (ih)PAS paradigm to target transcallosal motor circuits in healthy individuals, STDP was induced in both local and interhemispheric neuronal circuits [17] and correlated with improved motor skill performance [17, 18]. Such a neuromodulation approach may be effective in improving functional recovery poststroke when there is sufficient residual integrity of underlying motor pathways (e.g., corticospinal tract) to benefit from neuromodulation strategies targeting interhemispheric pathways [13]. However, STDP induction with ihPAS has not been investigated in stroke and its potential impact on neuroplasticity, arm motor skill performance, and learning in individuals with chronic stroke-related arm impairment is unknown.

In this early-stage clinical trial protocol, we will explore ihPAS as a novel noninvasive brain stimulation approach to induce STDP in interhemispheric motor connections to transiently modify IHI and ipsilesional cortical excitability targeting cortical pathways implicated in mediating chronic stroke-related arm motor impairment. The aims of the trial are to: (1) characterize STDP induction in intracortical and transcallosal circuits by ihPAS and (2) investigate the effect of ihPAS on motor skill acquisition and learning. We hypothesize that ihPAS of transcallosal connections between M1_HAND_ representations will: (1) induce STDP that increases cortical excitability of the targeted region (iM1_HAND_) and reduces abnormal IHI from cM1_HAND_ to iM1_HAND_ and (2) enhance motor skill performance and learning of a modified serial reaction time task (SRTT) after STDP induction.

## 2. Methods

Twenty individuals with subcortical stroke aged 40–85 in the chronic stage of recovery (>6 months) with mild–moderate impairment of the paretic arm (Fugl-Meyer upper extremity motor score [19] between 30 and 60 out of 66) will be recruited. The stage of recovery was selected to mitigate potential confounding influences of spontaneous biological recovery and/or effects associated with concurrent rehabilitation services. Inclusion criteria were individuals aged 40–85 with a first-time middle cerebral artery stroke affecting the corona radiata or internal capsule will be recruited. Stroke in these regions is common, comprising 34% of all strokes [20]. Additionally, a discernable MEP within the paretic FDI muscle is required to confirm the functional integrity of the primary corticospinal pathway for a participant to be included in the study. Exclusion criteria of the participants were as follows: (1) if they are outside the age range of 40–85; (2) if they show signs of dementia (score < 20 on the Montreal Cognitive Assessment) [21]; (3) if they have aphasia (score < 13 on the Frenchay Aphasia Screen) [22]; (4) if they have a history of head trauma, a major psychiatric diagnosis, a neurodegenerative disorder, or substance abuse; or (5) if they report contraindications to TMS or magnetic resonance imaging(MRI).

**Figure 1:**
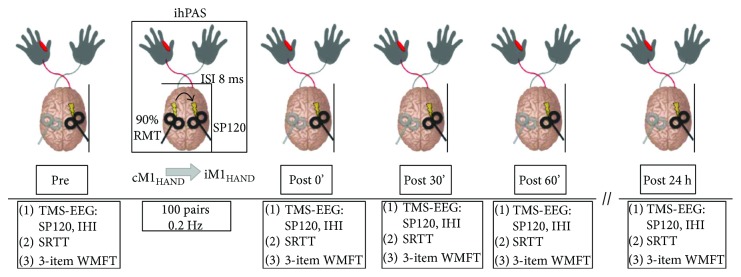
Experimental design for ihPAS visits (active and control conditions). The effect of ihPAS on (1) iM1 cortical excitability (TMS-EEG), (2) paretic arm motor skill performance (SRTT), and (3) motor function (3-item WMFT) will be evaluated. See associated sections below for additional details. Single (1 mV) and paired pulse (SICI, ICF, and IHI) TMS-EEG will be performed immediately prior to (pre) and at three time points (post 0′, post 30′, and post 60′) following ihPAS. Contralesional-to-ipsilesional M1_HAND_ ihPAS (cM1_HAND_-to-iM1_HAND_) will be delivered at 0.2 Hz with 100 stimulus pairs each separated by 8 ms. The “post 24 h” will be used to assess delayed effects of ihPAS. ihPAS visits for the active and control conditions will be separated by at least one week.

Participants will complete a total of 5 visits to the laboratory. First, enrolled participants will complete a visit where they will complete a magnetic resonance imaging (MRI) scan and baseline assessments of cognition, upper extremity physical impairment, motor function, and hand dexterity. Next, enrolled participants will complete two sessions of ihPAS, each with a 24-hour retention test, over 2 weeks. Assessments of cortical excitability using electromyography (EMG) and electroencephalography (EEG) of TMS-evoked cortical responses and motor skill performance will be conducted before ihPAS and then at three time points following ihPAS to evaluate the immediate time course of effects (Figure 1). Following each ihPAS session, the participant will return for a 24-hour retention test the following day where neurophysiologic and motor skill performance testing will be completed. Each ihPAS session will be separated by approximately one week, and the stimulation condition (active versus control ihPAS condition) will be randomized before the first ihPAS session. Both the experimenter holding the TMS coils and participant will be blinded to stimulation condition. The experimenter holding the coils during ihPAS will not have knowledge of the stimulation condition and will wear ear protection to minimize auditory information that could identify the stimulation condition. The participants will wear active noise-emitting earplugs to mask auditory cues of the stimulation condition. A separate experimenter will adjust the stimulation parameters. During offline data processing and analysis, the individual viewing the data will be blinded to ihPAS condition until final statistical analyses are performed. A questionnaire will be administered after each ihPAS visit to screen for potential side effects associated with stimulation (e.g., headache and neck pain).

### 2.1. MR Acquisition

A high-resolution T_1_ scan (TR = 7.4 ms, TE = 3.7 ms, flip angle *θ* = 6°, FOV = 256 mm, 160 slices, 1 mm thickness, scan time = 3.2 min) will be performed on a Siemens Trio TIM 3T whole-body MRI scanner housed within the Center for Systems Imaging, a core facility at Emory University. The study team will have direct and immediate access to the data using Emory University's secure network server.

### 2.2. Clinical Assessments of Motor Behavior

Arm motor behavior will be evaluated by a licensed physical therapist during the first visit with the Wolf Motor Function Test (WMFT), shown to be a valid and reliable test of motor function in stroke [23], the upper extremity portion of the Fugl-Meyer Assessment [19] to index paretic arm impairment, the Nine-Hole Peg Test (NHPT) [24] to assess hand dexterity, and hand-held dynamometry to measure grip strength [25]. These assessments will be used in secondary exploratory analyses to characterize relationships between baseline motor behavior and intervention response.

### 2.3. TMS Procedures

Participants will be seated comfortably in an adjustable reclining chair for all procedures. To reduce within- and between-session variability, the coil location and trajectory for the M1 stimulation site will be recorded and monitored in real-time with a stereotactic neuronavigation system (BrainSight®, Rogue Research Inc.) using the T1-weighted anatomical scans acquired for each participant. TMS will be delivered using a 70 mm hand-held figure-of-eight coil over the iM1 and a 50 mm branding iron coil over the cM1. Each coil will be connected to a monophasic stimulator (200 [2], Magstim Company Ltd.).

Stimulation will initially be localized over the M1 representation corresponding to the target muscle (first dorsal interosseous (FDI)) using navigated TMS and evoked responses in the FDI will be measured with EMG (BrainAmp ExG amplifier, Brain Products GmbH) (sampling rate: 5 kHz, frequency range: 0.53–499 Hz, 16 bit, range: −16.384–16.384 mV). Resting motor threshold (RMT) (% of maximum stimulator output) will be determined bilaterally using standard protocols [26]. If an MEP cannot be elicited at rest in the paretic FDI, then the active motor threshold (AMT) of iM1 will be determined. Maximum voluntary contraction (MVC) of the FDI during isometric abduction of the second digit will be measured. During AMT determination, a low-level (10% maximum voluntary contraction (MVC)) isometric contraction of the FDI will be performed with visual feedback displayed on a computer screen in front of the participant. If an MEP cannot be elicited at rest or when the paretic FDI is active, then the participant will be excluded from the study. The stimulator will be set to 120% of the MT (SP120) during TMS assessments. All TMS procedures will be performed bilaterally (iM1 and cM1). All stimulation parameters will fall within published safety guidelines [27].

### 2.4. ihPAS Intervention Protocol

One hundred pairs of pulses will be delivered over cM1_HAND_ and iM1_HAND_ at a frequency of 0.2 Hz for ~8.3 min [28] with subthreshold (90%RMT) cM1_HAND_ stimulation preceding suprathreshold (SP120) iM1_HAND_ stimulation at a fixed interpulse interval of 8 ms [17]. This interpulse interval elicits consistent and robust IHI when applied nonrepetitively [29]. When applied repetitively, increased cortical excitability in the targeted region (right M1_HAND_) and decreased IHI from left M1_HAND_ onto right M1_HAND_ have been observed in healthy individuals [17]. Control ihPAS procedures will be identical except the interpulse interval will be set to 1 ms causing asynchronous stimulus arrival in iM1 to avoid STDP induction. This paradigm is an effective control intervention to evaluate the effects of ihPAS-induced STDP [17]. The order of performing the ihPAS conditions (active versus control) will be randomized for each participant to minimize potential order effects.

### 2.5. TMS Assessments of Cortical Excitability

Single TMS suprathreshold (SP120) pulses will be delivered over the M1 representation for the FDI (frequency jittered from 0.1–0.25 Hz) while the participant is seated quietly with eyes open and FDI at rest to assess general levels of corticospinal excitability [30]. IHI will be evaluated using bifocal TMS with a suprathreshold (SP120) conditioning pulse delivered over the contralateral M1 FDI representation 10 ms prior to the test pulse (intensity: SP120) over the homologous region of M1 [29]. TMS assessments of local and interhemispheric cortical excitability and connectivity will be performed bilaterally. The testing order of each hemisphere will be randomized across participants and held constant across assessment time points for each individual participant.

For each TMS assessment, 30 pulses will be delivered to maximize within- and between-session reliability [31] and stored for offline data analyses. Peak-to-peak MEP amplitude for the single pulse (MEP_SP120_) will be the primary dependent measure of local iM1 excitability. For the IHI condition, MEP ratio (conditioned MEP/unconditioned MEP) will be calculated as the primary outcome measure characterizing IHI.

### 2.6. EEG Recording of TMS-Evoked Cortical Responses

Electroencephalography (EEG) data will be recorded during all TMS assessments. TMS-evoked cortical responses can be used to directly assess intracortical neuronal circuit excitability and connectivity between distinct cortical regions [32–34]. Electroencephalography data will be collected using a 32-channel TMS-compatible electrode cap (Easy Cap) and amplifier (BrainAmp DC, Brain Products Ltd.). Signals will be collected continuously (sampling frequency: 5000 Hz, impedance: <5 kΩ, frequency range: 0–1000 Hz, 0.5 *μ*V/bit resolution) during TMS assessments of cortical excitability. TMS-induced auditory artifacts will be reduced by using earplugs and frequency-specific noise to mask the audible TMS coil click [35]. Correction for residual artifacts will be conducted during data analysis.

### 2.7. EEG Assessment of Interhemispheric Cortical Connectivity

TMS-evoked EEG responses will be used to quantify abnormal interhemispheric connectivity after ihPAS intervention via imaginary phase coherence analysis (as detailed in [36]). All data preprocessing will be performed in EEGLAB, a MATLAB-based, open-source, freely available software environment [37]. EEG data will be resampled (1000 Hz), filtered (0.3–100 Hz), and average rereferenced. Automatic rejection of continuous data containing large artifacts will be performed. Data epochs (−1000 to 4000 ms with respect to TMS delivery) will be extracted for subsequent imaginary phase coherence analysis (as detailed in [36]). Post-TMS coherence values between electrodes overlying M1 bilaterally (C3 and C4) will be calculated within the beta frequency range (15 to 30 Hz). Changes in beta coherence over time will be expressed relative to the prestimulus interval using custom-built MATLAB functions [33]. TMS-evoked beta coherence during the IHI from cM1 onto iM1 condition will be the primary dependent measure of effective interhemispheric connectivity.

### 2.8. Behavioral Assessment

We will assess motor skill performance of each participant using a serial reaction time task (SRTT). Paretic arm motor function will be evaluated on a 3-item abbreviated version of the WMFT. Each assessment will be performed at each time point (Figure 1).

#### 2.8.1. SRTT Paradigm

**Figure 2:**
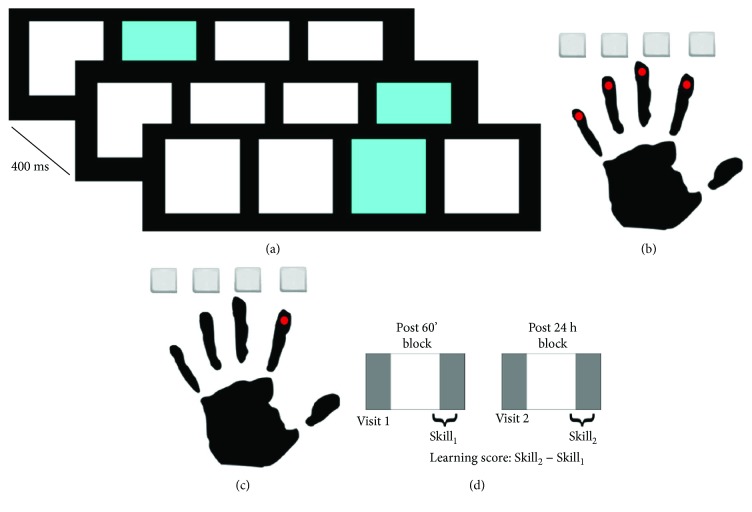
Modified serial reaction time task (SRTT) design. (a) Visual target (blue) presented 400 ms after each correct key press made either (b) with each finger or (c) just the second digit depending on degree of digit individuation during task performance. (d) Repeated trials (white) sandwiched between random trials (gray) for each block. *Skill* is defined as difference in RT between last 50 repeated trials and final random trials. *Learning* is defined as the difference between skill_1_ at post 60′ and skill_2_ at post 24 h.

Participants will perform a modified version of the SRTT [38] while seated comfortably in the same chair used for TMS procedures. Four equally sized and spaced squares will be displayed on a tablet touch screen (Acer Iconia One 10, screen size: 10.1″, screen resolution: 1200 × 800 pixels) positioned in front of the participant (Figure 2). Squares will be 160 × 160 px in size. Squares will be equally spaced at 70 px [39]. If participants are unable to adequately individuate finger movements, then they will perform the task using the second digit for all key presses. Participants will be instructed to press the square corresponding to the target square when the target is highlighted. Response time (RT) will be calculated as the time difference between target highlight and correct response. Key press accuracy will also be recorded and stored for secondary analyses. Accuracy values on the SRTT tend to asymptote above 90% early after initial task exposure in healthy individuals. Each SRTT block will consist of trials of both repeated and random sequences of order presentation. Fifteen repeats of a 12-element sequence (180 trials) will be preceded and followed by 50 trials (100 trials total) presented in a random order for a total of 280 key presses in each practice block [40].

### 2.9. Behavioral Analysis of SRTT Skill and Learning

Skill will be defined as the difference between the average RT during the last 50 sequential trials and average RT during the second set of random trials within each block [40] (Figure 2(d)). To evaluate the effect of ihPAS on motor skill acquisition, skill during the three post-ihPAS assessments will be compared to pre-ihPAS Skill. A learning score will be calculated as the difference in skill between post 60′ and post 24 h.

### 2.10. Abbreviated (3-Item) WMFT Procedure

Due to time constraints of the experimental design, three items of the WMFT will be used to evaluate functional motor performance at each assessment time point. Streamlined versions of the WMFT have been shown to be useful in evaluating poststroke motor function across the continuum of recovery [41, 42]. The 3 items were selected based on task difficulty ranging from easiest (hand to table) to most difficult (stack checkers) along with a task of moderate difficulty (lift can) [43]. Each task has different control demands and number of actions required to complete successfully. Task performance will be timed (maximum time: 120 s) and rated using the Functional Ability Scale (FAS) with ratings from 0 (no use) to 5 (normal use). A task rate (60 s/performance time) will be calculated for each task and the mean task rate across the three tasks will be the primary dependent measure of motor function used in a secondary analysis of how ihPAS may affect paretic arm motor function. Task rate is a valid and sensitive measure of hemiparetic motor function that better approximates a normal distribution than typical median task completion times when using the standard WMFT [44].

## 3. Discussion

Stroke is the leading cause of adult disability in the U.S. and its prevalence is expected to rise by 20% over the next 20 years, with tripled stroke-related costs [45]. Innovations in acute stroke management have reduced mortality rates, leaving more individuals with rehabilitation needs that will go unmet unless similar innovations in stroke rehabilitation strategies occur. New treatment approaches founded on empirical evidence and sound theoretical models are urgently needed to improve rehabilitation outcomes for a steadily increasing number of stroke survivors living with persistent disability. The present study will investigate the application of a novel noninvasive brain stimulation approach to induce neuroplastic changes in the brain of stroke survivors living with persistent impairment of the upper limb. If adaptive neuroplasticity underlies the learning of new movements critical to recovery after stroke, then approaches that can augment the brain's natural capacity for neuroplasticity have the potential to improve recovery. This study protocol will evaluate the effects of targeting connections between the primary motor cortices that are critical to normal movement. After stroke, the activity mediated by these connections is commonly disrupted, leading in many cases to maladaptive neuroplastic changes associated with impaired movement. By noninvasively inducing neuroplasticity of these specific connections, we can study the behavioral effects on motor skill performance and learning through use of this protocol. In addition, by using an innovative stimulation-measurement (TMS-EEG) corecording technology, we can directly evaluate brain responses to noninvasive brain stimulation and study the causal effects of brain activity in one area on activity in another area to better understand brain network properties of stroke recovery.

Noninvasive brain stimulation (NIBS) using repetitive activation of circumscribed brain regions with magnetic or electrical stimulation has shown promise as a potential alternative and/or augmentative therapeutic approach to traditional physical therapy after stroke with few side effects [46, 47]. However, the effectiveness of noninvasive brain stimulation remains unclear despite intensive inquiry over recent years [12, 48]. A recent review proposed that refining and developing NIBS approaches and updating models have the potential to lead to broader clinical implementation of promising NIBS techniques [13]. This scientifically based and clinically relevant study will incorporate these recommendations by evaluating a novel NIBS approach and by further studying the value of the interhemispheric competition model of stroke recovery. Furthermore, we will be performing retrospective analyses to investigate the effect of structural reserve of salient cortical structures using additional neuroimaging data being collected on study participants prior to the intervention. These additional exploratory analyses will help to identify characteristics of individuals who may show the greatest responses to the ihPAS neuromodulatory approach. If ihPAS demonstrates the ability to produce a significant neuromodulatory effect and/or enhance motor performance, then future studies could compare the clinical efficacy of ihPAS versus established neuromodulation approaches (e.g., rTMS or TES). The results from this fundamental but essential study will inform the design of future studies focused on modulating and measuring brain behavior to restore arm function after stroke.

Taken together, ihPAS may offer an innovative and promising neuromodulatory approach to target mechanisms fundamental to stroke recovery requiring systematic investigation. The proposed project will be the first step in determining the clinical utility of ihPAS to improve functional outcomes after stroke. If successful, ihPAS could provide a novel neuromodulation technology to augment current clinical rehabilitation strategies to improve stroke outcomes. Regardless of the outcome, findings from this study will provide important information for refining or reconceptualizing neurobiologically based models of persistent arm impairment after stroke. The principal long-term objective of this work is to conclusively define the salient neuroplastic signatures of stroke recovery that can be leveraged into improved therapeutic outcomes and quality of life for each stroke survivor.

## Additional Points

*Trial Status*. Participant recruitment is still ongoing.
